# Prognostic Value of Serum Tumor Markers in Medullary Thyroid Cancer Patients Undergoing Vandetanib Treatment: Erratum

**DOI:** 10.1097/01.md.0000479949.09661.21

**Published:** 2016-01-22

In the article “Prognostic Value of Serum Tumor Markers in Medullary Thyroid Cancer Patients Undergoing Vandetanib Treatment”,^1^ which appeared in Volume 94, Issue 45 of *Medicine*, funding information was originally omitted. The article has since been corrected online.

## References

[1] Werner RA Schmid JS Muegge DO Prognostic value of serum tumor markers in medullary thyroid cancer patients undergoing vandetanib treatment. *Medicine* 2015 94:e2016. 26559299 10.1097/MD.0000000000002016 PMC4912293

